# Regulatory T cell metabolism at the intersection between autoimmune diseases and cancer

**DOI:** 10.1002/eji.201948470

**Published:** 2020-10-26

**Authors:** Henry Kurniawan, Leticia Soriano‐Baguet, Dirk Brenner

**Affiliations:** ^1^ Experimental and Molecular Immunology Department of Infection and Immunity Luxembourg Institute of Health Esch‐sur‐Alzette Luxembourg; ^2^ Immunology and Genetics Luxembourg Centre for Systems Biomedicine (LCSB) University of Luxembourg Belvaux Luxembourg; ^3^ Faculty of Science Technology and Medicine University of Luxembourg Esch‐sur‐Alzette Luxembourg; ^4^ Odense Research Center for Anaphylaxis Department of Dermatology and Allergy Center Odense University Hospital University of Southern Denmark Odense Denmark

**Keywords:** autoimmunity, cancer, metabolism, regulatory T cells

## Abstract

Regulatory T cells (Tregs) are critical for peripheral immune tolerance and homeostasis, and altered Treg behavior is involved in many pathologies, including autoimmunity and cancer. The expression of the transcription factor FoxP3 in Tregs is fundamental to maintaining their stability and immunosuppressive function. Recent studies have highlighted the crucial role that metabolic reprogramming plays in controlling Treg plasticity, stability, and function. In this review, we summarize how the availability and use of various nutrients and metabolites influence Treg metabolic pathways and activity. We also discuss how Treg‐intrinsic metabolic programs define and shape their differentiation, FoxP3 expression, and suppressive capacity. Lastly, we explore how manipulating the regulation of Treg metabolism might be exploited in different disease settings to achieve novel immunotherapies.

## Graphical abstract

Regulatory T cells exert immune tolerance and are critical for immune homeostasis. Tregs, which are implicated in autoimmunity and cancer, orchestrate a unique metabolism to maintain their integrity and function. Manipulating Treg metabolism may pave the way towards the development of novel immunotherapies.

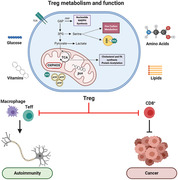

## Introduction

Regulatory T cells (Tregs) are a subset of CD4^+^ T helper (Th) cells that is indispensable for the maintenance of peripheral tolerance and immune homeostasis [1, 2]. Tregs account for ∼5–10% of total circulating CD4^+^ T cells. The majority of Tregs originate and mature in the thymus, constituting the thymus‐derived Treg population (tTregs). A fraction of the body's Tregs arises from naïve CD4^+^ T cells upon antigen stimulation and differentiates in the periphery, constituting the peripherally derived Treg population (pTregs) [3]. While both tTregs and pTregs have been linked to the establishment of peripheral tolerance, how each exerts its Treg suppressive function in various contexts remains to be further clarified.

The discovery of Forkhead box P3 (FoxP3) as the lineage‐specific master transcriptional regulator of all Tregs opened up new avenues for characterizing transcriptional regulation in this subset [4]. FoxP3 expression is essential for normal Treg identity and function [5, 6, 7]. Mutations in this gene result in severe immune dysregulation and initiate the autoimmune (AI) disease polyendocrinopahthy enteropathy X‐linked (IPEX) syndrome in humans as well as a severe, spontaneous autoimmune disorder in mice [8]. Tregs also express the α‐subunit of the IL‐2 receptor (CD25) and require constitutive IL‐2 signaling to maintain their homeostasis and function [9, 10]. The loss of CD25 in Tregs abrogates Treg development and FoxP3 expression, but not in mature Treg [11, 12].

It has become clear that the metabolic reprogramming that occurs in activated immune cells is essential for their proper functions, leading to the creation of a new field of research termed “immunometabolism” [13]. Recent studies have underlined the role of cellular metabolism in the generation and maintenance of various types of immune cells, including Tregs [14], and have revealed that distinct T cell subsets utilize different energetic and biosynthetic pathways to sustain their activities. Both human and murine naive CD4^+^ Th cells use oxidative phosphorylation (OXPHOS) for energy generation in the quiescent state. Upon activation by antigen, these cells proliferate and differentiate into CD4^+^ Th effector cells (Teffs), such as Th1, Th2, and/or Th17 cells and switch from OXPHOS to a highly glycolytic form of metabolism. In contrast, upon activation, Tregs employ glycolysis at a low rate and high lipid oxidation [15, 16]. This divergent use of metabolic pathways can influence cell fate in various ways. For instance, fatty acid oxidation (FAO) fosters the generation of Tregs while dampening the polarization of Teffs [15]. Researchers have therefore focused on investigating how cellular metabolism affects cellular functions in specific contexts. A key goal is to identify the metabolic checkpoints that interfere with cellular activity in the context of abnormal immune homeostasis.

In this review, we discuss the metabolic features of Tregs and delineate how the metabolic strictures shape Treg induction and function. In addition, we describe the relevance of these metabolic pathways in AI diseases and cancer, and explore how their manipulation might lead to new therapeutic avenues.

## Overview of metabolic control in Treg cells

Nutrients and metabolites are critical for normal immune cell function, and it is now well known that both under‐ and over‐nutrition can contribute to dysregulation of the immune system. However, the precise pathways modulating nutrients and metabolites so as to influence the functions of particular immune cell types remain obscure.

Tregs engage a nutrient‐sensing mechanism to adapt to both intrinsic and extrinsic environmental cues and trigger metabolic reprogramming to maintain their activity. These pathways of cellular metabolism are highly interconnected and critical for Treg function [17]. Tregs can select their usage of substrates and metabolic pathways to ensure their survival and function [18]. Under normal homeostatic conditions, where FoxP3 expression is maintained, the majority of Tregs are stable in phenotype and function. However, this stability depends on each cell's ability to prevent disruption of FoxP3 expression and the acquisition of an inflammatory effector function [10, 19]. In the context of pathologies, changes in nutrient availability or alterations to genes associated with metabolic regulation can affect Treg stability and function [20, 21, 22]. Managing the balance of these metabolic shifts is critical for the maintenance of Treg suppressive capacity. Of note, any difference in the metabolic regulation between tTreg and pTreg is still yet to be determined. Vitamins, glucose, lipids, and amino acids (AAs) are the major groups of metabolic substrates accessed by Tregs. Although each substrate is important for distinct functions and aspects of Treg regulation, they are closely interconnected and can be utilized simultaneously.

We will first discuss how the sensing of nutrients and metabolites can influence Treg function, and then highlight the signaling pathways that establish this metabolism‐dependent control of FoxP3 expression and other Treg‐specific characteristics.

### Vitamins that shape Treg activity

Vitamins are important regulators of Treg cell development, proliferation, and function. Among these, vitamins A, B_9_, C, and D have been the best studied and implicated in Treg biology [13].

#### Vitamin A

The metabolite all‐*trans* retinoic acid (ATRA), which is derived from vitamin A, promotes FoxP3 expression and thus Treg development. Upon the induction of TGF‐β signaling during murine Treg differentiation, ATRA stimulates signaling by extracellular‐related kinase (ERK1/2). This enhanced ERK signaling leads to increased histone methylation and acetylation of the FoxP3 promoter as well as the “conserved non‐coding DNA sequence” (CNS) element in this locus, without altering its DNA methylation [21, 23, 24]. FoxP3 expression is induced while pro‐inflammatory cytokine gene expression programs are suppressed. This ATRA‐induced stabilization of FoxP3 expression has also been observed in the cultures of expanding human Tregs during inflammation [25]. These findings have clinical relevance, since retinoic acid is superior to the mTORC1 inhibitor rapamycin in promoting stable FoxP3 expression and rapamycin is a drug widely used as an immunosuppressant for graft rejection in the clinic (Table 1) [26, 27, 28].

**Table 1:** Pharmacological inhibitors of the energy‐generating metabolic pathways and their regulators in Tregs, effects and diseases applied

Metabolic Pathway	Target	Drug	Effect on Tregs	Associated Disease	Species	Reference
Glucose metabolism	Glut‐1	CG‐5	Induce Treg differentiation	SLE	Mouse	[99]
	Glycolysis	2‐DG	Induce Treg differentiation and suppression	Experimental autoimmune neuritis	Mouse	[102]
				Skin and heart transplantation	Mouse	[105]
	PDHK	DCA	Increase Treg expansion	EAE	Mouse	[55]
	One‐carbon metabolism	Methotrexate	Increase Treg expansion	Rheumatoid arthritis, psoriasis, Crohn's disease and multiple sclerosis	Human	[39]
Lipid Metabolism	Mevalonate pathway	Statins	Dampen Treg stability and function			
	PPARγ (FA oxidation)	Pioglitazone	Induce VAT Tregs	Obesity	Mouse	[77]
	ACC (FA synthesis)	Soraphen A	Induce Treg differentiation	EAE	Mouse	[70]
	ACC (FA synthesis)	TOFA	Impair Treg proliferation	Glioblastoma	Mouse	[117]
	Lipid uptake	SSO	Impair Treg suppression			
Glutamine Metabolism	Glutamine uptake	DON	In combination with metformin and 2‐DG, promote Treg generation	Skin and heart transplantation	Mouse	[105]
Metabolic Regulators	mTORC1	Rapamycin	Increase Treg stability, generation and function	Graft rejection	Human	[26, 27]
				Autoimmune phenotype	Mouse	[61]
	AMPK	Metformin	Induce Treg differentiation	Type II diabetes	Human	[103]
				Skin and heart transplantation	Mouse	[105]
				Inflammatory bowel disease	Mouse	[103]
				EAE	Mouse	[104]
	mtROS	MitoTEMPO	Inhibit Treg damage and apoptosis	EAE	Mouse	[107]

SLE, Systemic lupus erythematosus; 2‐DG, 2‐Deoxy‐D‐Glucose; PDHK, Pyruvate dehydrogenase kinase; DCA, Dichloroacetate; EAE, Experimental autoimmune encephalomyelitis; SSO, Sulfo‐N‐succinimidyl oleate; TOFA, 5‐tetradecyl‐oxy‐2‐furoic acid, ACC: Acetyl‐CoA carboxylase; DON, 6‐diazo‐5‐oxo‐L‐norleucine; mtROS, mitochondrial reactive oxygen species.

#### Vitamin B9

The enzyme dihydrofolate reductase converts dietary vitamin B_9_, also known as folic acid (FAc), into tetrahydrofolate. Tetrahydrofolate is an essential precursor for one‐carbon metabolism (1CM), which is a metabolic cycle predominantly involved in DNA synthesis, methylation, and redox regulation. Tetrahydrofolate is methylated to generate 5‐methyltetrahydrofolate, which binds with high affinity to folate receptors to drive 1CM [29]. Tregs express high levels of folate receptor 4 (FR4), and FAc deficiency leads to colonic inflammation in mice. These data establish the importance of FAc and 1CM in sustaining Treg‐mediated suppression of inflammations *in vivo* [30].

#### Vitamin C

Vitamin C increases the induction and stability of murine FoxP3^+^ pTregs by promoting demethylation of the Treg‐specific demethylation region (TSDR), a conserved CpG‐rich region within the FoxP3 locus [31]. Interestingly, vitamin C seems to have differing effects on pTregs and tTregs. Oyarce et al. and others showed in mice that, whereas vitamin C did not affect tTreg suppressive capacity, it greatly enhanced the function in pTregs both *in vitro* and *in vivo* [32]. It is intriguing to speculate that this difference may be due to distinct modes of metabolic control in tTregs versus pTregs, an issue that needs to be further clarified. Kasahara et al. further showed that vitamin C promoted the induction of Treg differentiation from human naïve T cells, which was very stable even in the presence of IL‐6 *in vitro* [33].

#### Vitamin D

The vitamin D‐derived metabolite calcitriol increases CTLA‐4 and FoxP3 expression in human Tregs and also enhances their suppressive capacity [34]. In murine models of systemic lupus erythematosus (SLE), as well as in patients who suffer from this disease, vitamin D supplementation increases the percentage of Tregs and decreases levels of Th1 and Th17 cells, B cells, and anti‐dsDNA antibodies [35, 36]. The reduction in these inflammatory parameters corresponds to the amelioration of disease severity. Other work has shown that vitamin D can induce the generation of tolerogenic dendritic cells (DCs) to support pTreg generation, increase tolerogenic cytokines IL‐10 and TGF‐β, and attenuate the development of colitis in mice [37]. Despite these advances, however, the molecular mechanism by which vitamin D regulates function of Tregs is yet not clear.

### Glucose metabolism and signaling pathways controlling Tregs cells

#### General pathways of energy production in T cells

**Figure 1:**
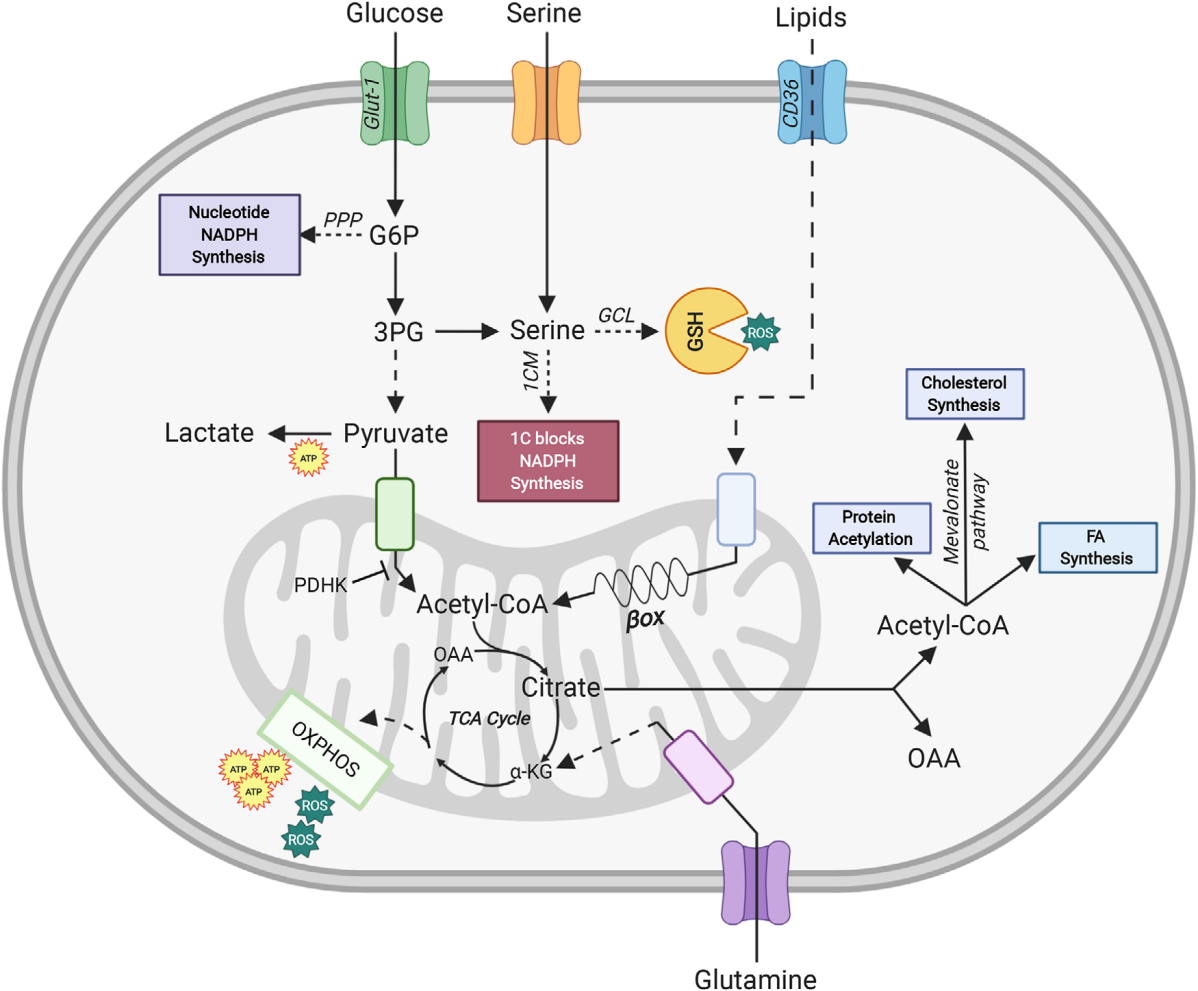
Schematic diagram of energy‐generating metabolic pathways and their regulators in Tregs. Upon TCR engagement and CD28 co‐stimulation, glucose enters the Tregs via the Glut‐1 transporter and is converted via glycolysis into pyruvate in the cytoplasm. Pyruvate enters the mitochondria and is converted into acetyl‐CoA, which drives the TCA cycle and thus energy production via oxidative phosphorylation (OXPHOS). OXPHOS is an efficient but slow route of cellular ATP production, and also generates ROS as by‐products. Under anaerobic conditions, cytosolic pyruvate is converted into lactate, generating less ATP. Depending on a cell's requirements, glucose entering the cell can also drive nucleotide and NADPH synthesis via the pentose phosphate pathway (PPP). Serine either enters the cell from the extracellular space or is synthesized from 3‐phosphoglycerate (3PG) generated from cytosolic glucose. This serine then either enters one‐carbon metabolism (1CM) to produce one‐carbon (1C) building blocks for anabolism, or generates the ROS scavenger glutathione (GSH). Lipids enter T cells via the CD36 transporter and then move to the mitochondria where the β‐oxidation (βox) of fatty acids (FA) is initiated. FA oxidation (FAO) generates acetyl‐CoA, which enters the TCA cycle to drive OXPHOS and provide energy. The TCA cycle metabolite citrate can be shuttled out of the mitochondria and converted back into cytosolic oxaloacetate (OAA) and acetyl‐CoA. The latter can be used for protein acetylation, cholesterol synthesis via the mevalonate pathway, and FA synthesis. Lastly, glutamine enters T cells, enters the mitochondria, and is converted into glutamate and α‐ketoglutarate (α‐KG). This α‐KG enters the TCA cycle to drive OXPHOS.

When a naive conventional CD4^+^ T cell experiences T cell receptor (TCR) engagement and CD28 co‐stimulation, the T cell uses the Glut1 transporter to take up glucose from the environment and initiate the glycolytic pathway [38]. This pathway converts glucose into various metabolites that can feed multiple branch pathways, such as the pentose phosphate pathway (PPP) that supports nucleotide production (Fig. 1). Glucose also feeds into serine biosynthesis plus 1CM to produce one‐carbon building blocks for macromolecule biosynthesis [39]. The final step of the glycolytic pathway is the generation of pyruvate, which either enters the TCA cycle in the mitochondria and is converted into acetyl‐CoA by pyruvate dehydrogenase (PDH), or remains in the cytosol and is converted into lactate by lactate dehydrogenase (LDH) [39]. Acetyl‐CoA within the TCA cycle reacts with oxaloacetate (OAA) to generate citrate that further fuels the TCA, the electron transport chain (ETC), and OXPHOS. OXPHOS is considered to be highly energetically efficient since it results in up to 36 molecules of ATP produced per molecule of glucose consumed [14, 39]. Alternatively, the citrate produced by the TCA cycle can exit the mitochondria and be converted back into cytosolic OAA and acetyl‐CoA by ATP‐citrate lyase (ACLY). Cytosolic acetyl‐CoA can be used for the synthesis of cholesterol or fatty acids (FA), or for protein acetylation (such as that of histones) to modulate gene expression [14, 39].

The generation of lactate from pyruvate in the cytosol was originally described as an inefficient energy pathway used predominantly during hypoxia due to the fact that only two ATP molecules are produced per glucose molecule consumed [14, 39]. However, activated Teffs use this route even under normoxic conditions, as it is the fastest way to generate energy and intermediate metabolites to build biomass for proliferation. This phenomenon was first described in cancer cells and is known as the Warburg effect [40, 41].

#### Signaling pathways controlling energy production in Tregs

**Figure 2:**
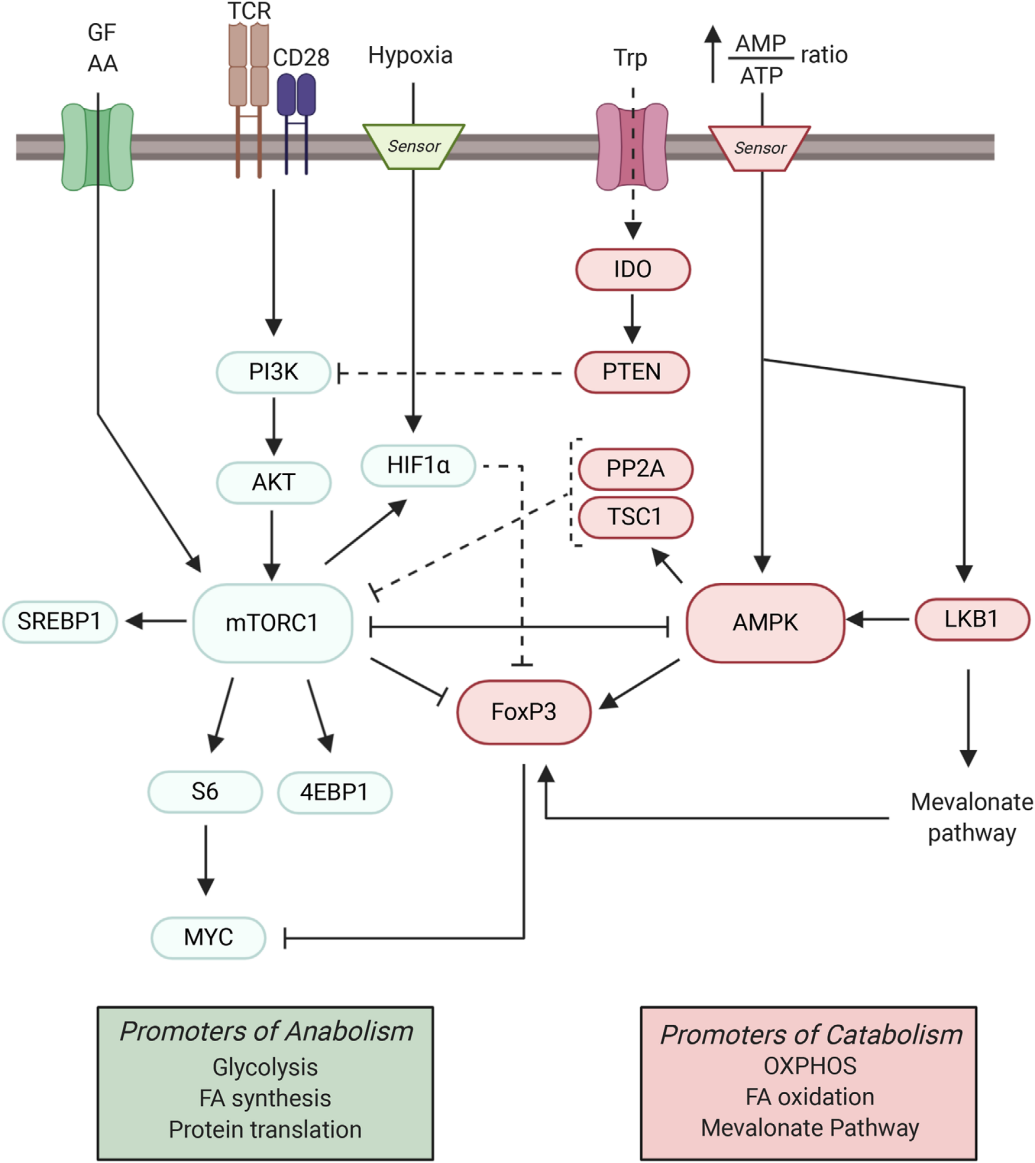
Schematic diagram of energy‐generating regulators in Tregs. The mTORC1 and AMPK signaling pathways are major metabolic checkpoints that modulate the FoxP3 expression and immunosuppressive activity of Tregs. Upon TCR engagement and CD28 co‐stimulation, the PI3K/AKT pathway is activated and induces mTORC1 signaling. The mTORC1 pathway can also be stimulated by the sensing of growth factor (GF) or amino acid (AA) availability. mTORC1 activation represses FoxP3 expression and thus Treg function. FoxP3 inhibits MYC, a downstream target of mTORC1, and so exerts direct metabolic control. FoxP3 is also downregulated by HIF1α, which is activated either directly by hypoxia or via mTORC1. HIF1α is therefore also a modulator of Treg function. Indoleamine 2,3‐dioxygenase (IDO), an enzyme involved in tryptophan (Trp) catabolism, activates PTEN. PTEN opposes PI3K‐dependent mTOR activation and thus supports Treg function. mTORC1 negatively regulates AMPK, which is a major promotor of catabolism and also regulates FoxP3 expression. AMPK is activated when energy supplies become low (high AMP/ATP ratio). LKB1 drives both AMPK activity and the mevalonate pathway, which is critical for Treg metabolism and function. Activated AMPK can also signal via TSC1 to inhibit the mTORC1 pathway. Likewise, protein phosphatase 2A (PP2A) is a negative regulator of the mTORC1 pathway.

In contrast to Teffs, both human and murine Tregs rely mostly on the oxidative pathway of glucose metabolism for their function in a manner tightly regulated by several key signaling cascades. An especially important pathway in this context is the PI3K/AKT/mTOR axis, which is activated in a T cell upon TCR engagement plus CD28 co‐stimulation [39]. Activated PI3K in turn phosphorylates AKT kinase, which leads to engagement of mTOR complex 1 (mTORC1) [42]. mTORC1 is the master regulator of anabolism and activates downstream targets such as S6 kinase and 4EBP1 to promote protein translation. mTORC1 also stimulates SREBP‐1 to trigger FA synthesis, and HIF1‐α to induce glycolysis (Fig. 2). mTORC1 is also activated by growth factors and nutrients, including the AAs leucine, arginine, and glutamine [39]. It has been shown that a lack of mTOR activity, or its specific inhibition by rapamycin, increases FoxP3 expression and causes naïve CD4^+^ T cells to differentiate into Tregs rather than Teffs both *in vitro* and *in vivo* [43, 44]. However, it has also been shown that mTORC1 is necessary for the suppressive function of Tregs, since *in vivo* deletion of raptor, a central component of mTORC1, specifically in mouse Tregs causes loss of Treg suppressive capacity, spontaneous Teff activation, and a fatal inflammatory disorder [45, 46]. In addition, Kishore et al. have shown that mTOR‐induced glycolysis is required *in vivo* and *in vitro* for human and murine Treg migration to inflammatory sites via the PI3K‐mTORC2‐mediated pathway [47]. mTOR also regulates the glycolytic‐lipogenic switch important for Treg cell growth and proliferation [48]. Despite mTOR levels in Treg being relatively low compared to Tconv, this nutrient sensor is still crucial for Treg function [49]. Tregs have been shown to have a dynamic mTOR requirement throughout their activity. Early downregulation of mTOR, followed by an increase in mTOR activation is critical for Treg cell expansion to occur [50]. Thus, delicate balance of mTOR is critical for normal Treg activity, and dysregulation of mTOR activity impairs Treg function. These observations reveal the dual role of mTOR signaling in Tregs: on one hand, mTOR induction promotes Treg proliferation and migration; on the other hand, it dampens Treg suppressive capacity [22].

mTORC1 signaling in T cells is negatively regulated by AMPK‐dependent signaling [39]. AMPK is a sensor of a cell's AMP/ATP ratio and becomes active when this energy balance is low (Fig. 2). AMPK signaling thus has the opposite effect on mTORC1‐responsive pathways, promoting mitochondrial OXPHOS and FA oxidation [42].

Negative regulators of mTOR critical for Treg function include protein phosphatase 2A (PP2A) and tuberous sclerosis 1 (TSC1) (Fig. 2). Genetic deletion specifically in murine Tregs of either of these two regulators impairs Treg suppressive capacity, resulting in spontaneous autoimmunity [51, 52].

The lipid phosphatase PTEN prevents PI3K activation and so is another inhibitor of the mTOR signaling pathway (Fig. 2). Consistent with other studies, ablation of PTEN in murine Tregs results in a spontaneous autoimmune lymphoproliferative disease [53]. PTEN‐deficient tTregs gradually lose CD25 and FoxP3 expression and increase glycolysis while reducing their mitochondrial fitness [53, 54]. In mice suffering from experimental autoimmune encephalomyelitis (EAE), a mouse model of human multiple sclerosis (MS), PTEN‐deficient Tregs are unable to suppress Teff functions, allowing autoreactive Teffs to attack the host's central nervous system [53].

HIF1α itself is also important in balancing Treg generation. HIF1α signaling induced by hypoxia blocks mitochondrial respiration and activates PDH kinase (PDHK), which inhibits PDH and so interferes with the conversion of pyruvate into acetyl‐CoA [39, 55]. It has also been reported that HIF1α promotes FoxP3 degradation by binding to this transcription factor, targeting it for ubiquitination and proteasomal degradation [56]. In line with these findings, HIF1α expression in murine T cells favors Th17 cell commitment over Treg generation *in vitro* [56].

#### FoxP3 expression in Tregs

As noted above, FoxP3 is the master transcription factor controlling Treg development. However, FoxP3 also regulates metabolism in mature Tregs by increasing their oxidative metabolism, catabolism, and OXPHOS (Fig. 2). Specifically, FoxP3 promotes the production of ETC proteins and enzymes involved in FAO, while simultaneously blocking PI3K‐AKT‐mTORC1‐driven glycolysis and anabolism [22, 57, 58]. In a low glucose/high lactate environment, FoxP3 is able to reprogram mouse Tregs to survive by blocking the anabolic mTORC1 target Myc and glycolysis, while increasing respiratory capacity via OXPHOS to increase the NAD/NADH ratio [39, 42, 57, 59]. OXPHOS is indispensable for Treg function since Treg‐specific ablation of Rieske iron‐sulfur protein (RISP) in mice, an element of ETC complex III, causes an autoimmune “scurfy‐like” disease [60]. Lastly, it was shown that increased Glut1 expression in murine Tregs enhances glycolysis and proliferation driven by mTORC1. The increased mTORC1 activity impairs tTreg and pTreg suppressive capacity and decreases FoxP3 expression both *in vitro* and *in vivo* [22, 61].

Collectively, current studies of energy metabolism in Tregs indicate that Treg proliferation and suppressive activity can be influenced by a variety of metabolic pathways, including anabolic signals driven by the mTORC1 axis and those arising from FoxP3‐dependent oxidative metabolism [22, 61].

### Lipid metabolism in regulatory T cells

#### Fatty acid oxidation

Lipid metabolism is essential for providing many cell types with an adequate supply of energy (Fig. 1) [62]. FA are a subgroup of lipids that have been implicated in human and murine Treg differentiation, survival, and function, although the exact contribution of the different lipids in both Treg population remain to be addressed [15, 55, 63]. Tregs recognize and take up FA via various receptors, including G protein‐coupled receptors (GPCRs), CD36, fatty acid‐binding protein (FABP), and fatty acid transport protein (FATP) [63, 64]. High levels of mitochondrial FAO then ensue in these Tregs to generate the energy needed to support their development [15]. In the absence of exogenous FA *in vitro* TGF‐β‐induced generation of FoxP3^+^ Tregs is abrogated in mice [15].

Several studies have indicated that FAO is critically needed to support Treg function [15, 55, 65, 66]. Carnitine palmitoyl transferase‐1 (CPT1) enzyme is essential for FAO. CPT1 attaches carnitine to long‐chain FA (LC‐FA) to facilitate LC‐FA transport from the cytosol into the mitochondria, where FAO occurs. Interestingly, Raud et al. showed that genetic deletion of CPT1a had no effect on the development or function of murine tTregs and pTregs [67], which is in stark contrast to the studies using the CPT1a inhibitor etomoxir [15]. When applied at higher concentrations the studies from Raud et al. and Divakaruni et al. indicated that etomoxir inhibits different targets beside CPT1a [67, 68]. However, it should be noted that Tregs can use CPT1a‐independent pathways to utilize fatty acids and that the translocation of short‐ or medium‐chain FAs does not involve CPT1a [66].

A balance between FAO and FA synthesis in Treg is determined by nutrient levels in the surrounding environment and intracellular energy status. Although Tregs primarily depend on FAO, they also require the balance between certain degree of *de novo* FA synthesis (which costs the cell energy) and FAO (which generates energy) [65, 69]. In contrast, Teffs are highly proliferative when functioning and require FA synthesis [70].

#### AMPK and LBK1

AMPK is part of a sensor system that is activated when a cell is in a low energy environment (Fig. 2). Activation of AMPK drives mitochondrial FAO while inhibiting *de novo* FA synthesis. To support their high demand for FAs, Tregs store substantial amounts of lipids within intracellular lipid droplets [71]. AMPK activation in Tregs stimulates mitochondrial oxidation of these lipids, while inhibiting *de novo* FA synthesis. Multiple studies have shown that tilting the balance towards lipid oxidation by modulating upstream targets of AMPK or pathways implicated in β‐oxidation *in vivo* promotes Treg development [70, 72, 73].

Liver kinase B1 (LKB1) is a kinase acting upstream of AMPK and another important metabolic sensor in Tregs (Fig. 2) [42]. Ablation of LKB1 in mice significantly reduces tTreg numbers and impairs their function. LKB1‐deficient Tregs lack functional mitochondria, abrogate FA oxidation and curtail OXPHOS, resulting in ATP depletion [72, 73].

LKB1 also modulates the mevalonate pathway that synthesizes the more complex lipids needed by Tregs. The mevalonate pathway produces cholesterol and the isoprenoid geranylgeranylpyrophosphate (GGPP), two metabolites that promote Treg proliferation and stability (Fig. 1 and 2) [74]. Blocking the mevalonate pathway *in vivo* via either ablation of HMG‐CoA reductase (HMGCR), a rate‐controlling enzyme in this pathway, or by pharmacological blockade using statins (Table 1), stimulates the conversion of murine Tregs into Th1‐like and Th17‐like Teffs and impairs tTreg suppressive capacity [74].

#### Acetyl‐CoA carboxylase and FA‐binding proteins

Acetyl‐CoA carboxylase (ACC) is an important enzyme for the initiation of FA synthesis. Blockade of ACC both *in vitro* and *in vivo* restrains the formation of Th17 cells and dampens IL‐17 production, but induces murine FoxP3^+^ Treg generation [70]. Likewise, FA‐binding proteins (FABPs) are pivotal for Treg suppressive function. Genetic or pharmacological inhibition of FABP5 leads to impaired lipid uptake and trafficking, which results in decreased FAO and OXPHOS, impaired lipid metabolism, and loss of murine pTreg suppressive capacity [66].

#### Dietary lipids

Dietary lipids are also linked to Treg biology, especially for Tregs in the colon (cTregs). Short‐chain fatty acids (SCFAs), such as butyrate, acetate, and propionate, are the major metabolites derived from bacterial fermentation of dietary fiber [63]. Several studies have shown that butyrate is involved in epigenetic modification by inhibiting histone deacetylases; in mice, exogenous butyrate increases histone acetylation of the FoxP3 locus, thereby supporting cTreg generation and stability [75, 76]. In addition, SCFAs can act via GPCR 109a to influence Tregs. The binding of butyrate to GPCR 109a on macrophages and DCs induces IL‐10 production, and this cytokine in turn promotes Treg generation in mice [76].

#### Peroxisome proliferator‐activated receptor‐γ

Peroxisome proliferator‐activated receptor (PPAR)‐γ is a regulator of lipid metabolism that has been associated with maintaining the homeostasis of Tregs in visceral adipose tissue (VAT). Interestingly, Treg‐specific deletion of PPAR‐γ in mice significantly abrogates the generation of Tregs in VAT but not in other organs [77]. Likewise, PPAR‐γ agonist pioglitazone (Table 1) upregulates expression of the FA transporter CD36 and increases FAO. These events result in the induction of VAT Tregs, which have positive suppressive effects on obesity‐associated metabolic disorders [77].

#### Polyunsaturated fatty acid

Long‐chain polyunsaturated fatty acid (PUFA), which is the preferred ligand for PPAR‐γ, has also been shown to shape the Th cell balance and the outcome of autoimmunity. Mice fed with PUFA show a significant reduction of Th1 and Th17 cells, inflammatory cytokines IFN‐ γ, IL‐17, IL‐6, TNF, and exhibit a higher number of Tregs [78]. Moreover, these mice have lower incidence of type 1 diabetes. A similar study using fat‐1 transgenic mice expressing *Caenorhabditis elegans* fat‐1 gene, which led to accumulation of PUFA, showed that these mice had a higher induction of Treg cells *in vitro* and *in vivo*, and lower clinical arthritis score [79].

Lipid metabolism clearly plays a critical role in Treg homeostasis, but further study is required to clarify exactly how LC‐FAO supports Tregs as well as the balance between FAO and FA synthesis in Treg biology. It is also possible that short‐ or medium‐chain FA may regulate Tregs differently than LC‐FA. These issues remain to be addressed.

### Amino acid metabolism in Tregs cells

#### AA transporters

Amino acids serve not only as a source of energy but also provide building blocks for protein and nucleic acid biosynthesis. There are two types of AAs: essential AAs, which are obtained from the diet, and non‐essential AAs, which can be synthesized by the host [80]. Amino acids that are not synthesized have to be imported into cells via AA transporter proteins. A single AA transporter can often facilitate the transport of multiple AAs, raising the complexity of their regulation [81]. Tregs express various AA transporters, with Slc7a5 being the most extensively studied. Slc7a5 interacts with Slc3a2 to form a large neutral AA transporter called LAT1 [82]. The regulation of AA transport via LAT1 directly influences mTOR activity [83, 84]. Given the prominence of mTOR in regulating Treg metabolism, additional study of other AA transporters and their effects on Treg function is warranted.

#### Branched‐chain AA and arginine

The branched‐chain AAs (BCAAs), leucine, isoleucine, and valine play a particularly important role in the metabolic reprogramming that supports Treg maintenance. Mice fed on a diet containing reduced BCAA exhibit decreased numbers of FoxP3^+^ Tregs in the periphery [85]. Similarly, genetic deletion of the BCAA transporter Slc3a2 specifically in murine Tregs abrogates their mTOR activity, resulting in loss of tTreg numbers and function, and thus spontaneous inflammation in the mutant mice [85]. In addition, a recent study has shown that Tregs cultured in the presence of arginine and leucine show sustained mTOR activity upon TCR stimulation. The absence of either AA in murine Tregs result in a reduced number of activated Tregs and these mice develop fatal autoimmune disease [86]. Lastly, in human CD4^+^CD25^+^FoxP3^+^ Tregs, exogenous arginine induces DNA hypomethylation rather than histone modification of the IL‐10 promoter, which leads to higher IL‐10 production by these cells [87].

#### Kynurenine and glutamine

Kynurenine is a product of tryptophan catabolism that is generated by IDO and is important for murine Treg generation, expansion, and suppressive function [88]. IDO inhibits mTOR signaling via PTEN and thereby inhibits Teff proliferation while promoting Treg induction (Fig. 2) [17].

Glutamine fuels the TCA cycle via glutaminolysis and is critical for reprogramming Teff metabolism upon activation (Fig. 1). Accordingly, glutamine depletion blocks Teff proliferation and cytokine production [89]. Perhaps surprisingly, depending on the model used, glutamine has an opposing effect on Tregs. *In vitro*, glutamine restriction shifts murine naïve T cell differentiation under Th1 polarizing conditions toward FoxP3^+^ pTregs [90]. However, *in vivo* study using an acute graft‐versus‐host disease (GVHD) mouse model showed that glutamine supplementation markedly increased the proportion of Tregs and inhibited GVHD‐induced inflammation in the intestine, liver, skin, and spleen [91]. Thus, the role of glutamine in Treg homeostasis needs to be further clarified.

#### Serine

Our group has recently shown that serine metabolism and redox regulation are intimately linked to Treg function. Serine feeds into the synthesis of glutathione (GSH), a major cellular antioxidant [92]. GSH synthesis also depends on the catalytic subunit of glutamate cysteine ligase (*Gclc*). Conditional deletion of *Gclc* in murine Tregs impairs their production of GSH [61]. These mutant Tregs consequently display increased levels of ROS but no apparent defects in both tTreg and pTreg homeostasis, stability or differentiation. However, *Gclc*‐deficient Tregs do show increased proliferation, activation, glycolysis, and OXPHOS, and the mutant mice develop spontaneous autoimmunity [61]. Importantly, *Gclc*‐deficient Tregs show elevations in both their synthesis and uptake of serine. Accordingly, dietary intervention in the form of feeding the mutant mice on serine/glycine‐deficient chow prevents the onset of autoimmunity. In line with this finding, dampening of serine metabolism reinstated the suppressive function of Tregs, firmly linking serine metabolism to Treg function [61].

The high serine levels in *Gclc*‐deficient Tregs lead to elevated mTOR activity, which in turn reduces FoxP3 expression and so Treg function [61]. Interestingly, this increase in mTOR activity in *Gclc*‐deficient Tregs stands in striking contrast to the opposite effect seen in *Gclc‐*deficient conventional T cells [93], and indicates that GSH exhibits subset‐specific functions. In Tregs, GSH controls a feedback loop between GSH and serine availability that controls Treg suppressive capacity [61].

Collectively, these observations clearly establish that a delicate balance of forces within AA metabolism determines the functionality of Tregs.

### Distinct metabolic regulation of human Tregs

The metabolic characterization of Tregs has been mainly focused on murine Tregs. Studies on the metabolic control of human Treg cells are still sparse. To complicate matters, it is well accepted that the human Treg population is highly heterogeneous. Mass cytometry analysis of circulating human Tregs identify over 22 subsets, depending on the expression level of surface markers such as CD45RA, CCR4, CD39, CD127, HLA‐DR, and Foxp3 [94]. Because of this and the lack of detailed information on each subset, we only discuss the common human Tregs subset that highly express CD25, Foxp3, and low expression of CD127.

There are many commonalties in the metabolic regulation of human and mouse Tregs, however, striking differences have been observed as well. Unlike murine Tregs that are low in glycolysis and high in FAO, human tTregs are highly glycolytic and show high mTOR activity [95]. Along the lines, *in vitro* proliferating human Tregs express high levels of genes linked to glycolysis (similar to murine Tregs) and FAO (different to murine Tregs), such as mTOR and AMPK. Similar to murine Tregs, human Tregs rely on a balanced metabolism, but the molecular consequences are different. In human Tregs blocking glycolysis with the inhibitor 2‐DG leads to differential expression of human FoxP3 splicing variants, which support their suppressive function [96]. Unrestrained glycolysis impairs human as well as murine Treg function. In human Tregs, the glycolytic enzyme Enolase‐1 has been shown to bind to the FoxP3 promoter and the CNS2 region. Enolase‐1 then represses the transcription of the isoform FoxP3‐E2, which is important for Treg mediated suppression [96].

Our group has shown that human pTregs also require well‐adjusted AA metabolism for their optimal function. Improper redox balance upon *Gclc* inhibition in human pTregs increases serine metabolism that consequently impairs FoxP3 expression and Treg function. Restoring a balanced serine metabolism can reestablish FoxP3 expression in human pTregs as well as in murine Tregs [61].

## Modulating Treg metabolism as a therapy for autoimmune diseases: Lessons learned from murine models

One of the most important aspects of the immune system is its ability to differentiate between foreign and self‐antigens. Autoimmunity arises when the immune system loses its tolerance to self‐antigens and starts to attack host tissues expressing these antigens. Autoimmune (AI) diseases affect ∼7–9% of the general population, with women being at higher risk than men. AI diseases are divided into two categories according to the extent of the affected tissue. Organ‐specific AI diseases, such as MS and inflammatory bowel disease (IBD), affect primarily one organ or system. Systemic AI diseases, such as SLE and rheumatoid arthritis (RA), disrupt homeostasis widely throughout the body. In all AI diseases, Th cells play an important role, either as disease contributors or as rescuers attempting to mitigate inflammation [64]. Autoreactive Teffs attack self‐tissues and cause AI disease, whereas Tregs, with their ability to shut down Teffs, are important for preventing AI disease [97, 98]. Therefore, enhancing the immunosuppressive activities of Tregs by modulating their cellular metabolism could represent a potential new therapeutic strategy for the treatment of AI diseases.

### CG‐5 and 2‐DG

As previously discussed, Tregs rely mostly on the oxidative metabolism of lipids and minor amounts of glucose to generate energy and maintain their suppressive capacity. In contrast, Teffs employ glycolysis and FA synthesis to sustain their effector functions [14, 42]. Therefore, drugs that favor the oxidative metabolism of Tregs and/or block the anabolic metabolism of Teffs could have a beneficial impact in the context of AI. For example, in both spontaneous and induced mouse model of SLE, reducing glucose intake using the Glut1 inhibitor CG‐5 (Table 1) ameliorated AI disease symptoms. *In vitro*, CG‐5 treatment of naive murine Th cells promoted Treg differentiation while blocking Th1 and Th17 polarization [99].

Another example is treatment with 2‐deoxy‐d‐glucose (2‐DG), which is a known inhibitor of glycolysis because it blocks the generation of glucose‐6‐phosphate (Table 1). Treatment of mouse naïve cells with 2‐DG*in vitro* limits Th17 generation while inducing Treg differentiation [100]. Our group has recently shown that, if ROS are uncontrolled in tTregs and pTregs these cells increase glycolysis at the cost of their suppressive activity, which causes severe autoimmunity [61]. Accordingly, we found that 2‐DG‐mediated glycolytic restriction was able to reverse the functional deficit of the mutant Tregs *in vitro* [61]. Others have shown that blocking glycolysis with 2‐DG*in vitro* reduces ATP levels, increases the AMP/ATP ratio, and thus activates AMPK signaling [101]. AMPK promotes catabolic reactions such as the oxidation of glucose and lipids, which are the pathways preferentially used by Tregs. Liu et al. showed that 2‐DG‐treated mice with experimental autoimmune neuritis, which is a mouse model of human Guillain‐Barré syndrome, blocked disease initiation and progression. 2‐DG promoted Treg differentiation while inhibiting that of Th1 and Th17 cells [102].

### Metformin

Metformin is another drug that promotes Treg differentiation and is already widely used in the clinic for the treatment of type II diabetes (Table 1) [103]. Metformin induces FAO through AMPK activation but also inhibits ETC complex I [42]. Studies have shown that the use of metformin in several mouse models of AI diseases, such as IBD and EAE, induces Treg generation while inhibiting Th17 differentiation. This skewing leads to a reduced disease burden in the metformin‐treated mice [103, 104]. These observations underline the importance of coordinating the balance between glycolysis and OXPHOS, a balance that is crucial for Treg suppressive capacity [61].

In addition to promoting Treg function, it can be advantageous to use metformin to help decrease the functions of self‐reactive Teffs. Glutaminolysis is a major pathway that is important for Teffs, less so for Tregs [90]. Lee et al. showed that a combination of metformin, 2‐DG and 6‐diazo‐5‐oxo‐l‐norleucine (DON), which is a glutamine antagonist (Table 1), blocked Teff proliferation and cytokine production in a mouse model of skin and heart transplantation [105]. Most helpfully, the same cocktail fostered Treg generation and so also promoted the tolerogenic response *in vivo*.

### Dichloroacetate

Another way of inducing oxidative metabolism of glucose is to inhibit the positive regulators of glycolysis. Dichloroacetate (DCA) blocks PDH kinase (PDHK), an inhibitor of PDH, and so maintains PDH in its active form (Table 1). Thus, the connection between glucose metabolism and the TCA cycle remains open in DCA‐treated cells and the oxidation of pyruvate in the TCA cycle is favored over its reduction into lactate. Gerriets et al. showed using the EAE model that mice treated with DCA presented with a lower disease burden due to increased Treg generation coupled with inhibition of Teff function [55].

### Rapamycin and HIF1α deletion

Teffs show highly active mTORC1. mTORC1 inhibition with rapamycin induces FAO, dampens Teff proliferation, and reinforces Treg generation [15, 101]. Rapamycin treatment of mice showing AI symptoms reverses their inflammatory phenotype, restores Treg functionality, and prolong the lifespan (Table 1) [61].

HIF1α has also been described as a key regulator in systemic and organ‐specific AI diseases, particularly SLE, IBD, and RA [42]. In the EAE mouse model, ablation of HIF1α leads to increased Treg differentiation and reduced Th17 development, which in turn dramatically decreases the AI disease score [56, 100].

### Methotrexate

In contrast to Tregs, Teffs rely on serine metabolism for macromolecule biosynthesis. Indeed, blocking this pathway with the chemotherapeutic drug methotrexate (Table 1) is used to treat patients with RA, psoriasis, Crohn's disease or MS in the clinic. This strategy induces Tregs while dampening Teff functions [39]. Again, these findings align with our demonstration that modulating serine metabolism, which is linked to a stress‐sensitive feedback loop in Tregs, controls Treg suppressive capacity, and is important for limiting autoimmunity in mice [61].

### Soraphen A

Tregs rely on lipid oxidation, while Teffs use FA synthesis. Therefore, activating FAO or inhibiting FA synthesis could be a therapeutic intervention for AI diseases. For example, ACC is the first enzyme in the *de novo* FA synthesis pathway [106]. *In vitro*, inhibiting ACC isoform 1 with the specific inhibitor Soraphen A (Table 1) induces Treg differentiation and impairs Th17 differentiation. In line with this result, Soraphen A administration*in vivo* attenuates EAE development in mice [70].

### Modulation of ROS

ROS is an important regulator of Treg function [61]. Our group has shown that disturbed redox signaling due to the genetic ablation of antioxidative GSH synthesis in murine Tregs leads to ROS accumulation. Increased ROS in Tregs skew the metabolic programs and lead to increased glycolysis, OXPHOS, and serine metabolism, which impaired Treg function and induce spontaneous autoimmunity [61]. In line with that, Alissafi et al. have recently shown that human and murine Tregs experience a specific metabolic reprogramming during autoimmunity [107]. Tregs from MS and SLE patients show elevated mitochondrial oxidative stress and a DNA damage response. Murine Tregs during EAE exhibited a similarly prominent mitochondrial ROS (mtROS) signature and ROS scavenging with mitoTEMPO reduced the disease burden (Table 1) [107].

Taken together, these data support the concept that fostering a metabolic state favorable to Treg function may be a promising strategy for the treatment of various AI diseases.

## Targeting Treg metabolism for cancer therapy

Incipient cancer cells evade immune recognition in a dynamic process of competition between immunosurveillance and tumor cell growth. Escaped tumor cells survive and are able to grow unchallenged, leading to cancer pathogenesis. Studies to delineate the mechanisms that promote adaptive anti‐tumor responses and exploit cancer cell vulnerabilities to restrain tumor growth are at the forefront of today's anticancer research [108]. The tumor microenvironment (TME) surrounding a growing malignancy is a complex and heterogeneous milieu that undergoes fluctuating changes in its physical and chemical properties [109]. For example, cellular signaling via HIF1α, extracellular acidity generated by the lactate released by both tumor and stromal cells, and metabolic competition for nutrients within the TME can all significantly alter the progress of tumorigenesis [48, 110, 111].

The contribution of Tregs to preventing/promoting tumor progression remains controversial. Tregs expressing unusually high levels of FoxP3 have been associated with improved patient survival in several cancer settings, including colorectal and esophageal malignancies [112]. However, it has become evident that, in most solid tumors, Tregs are a negative player and detrimental to robust antitumor immunity [111, 113, 114]. Most Tregs within the TME exhibit an increase in suppressive capacity and produce signaling molecules that disable antitumor immunity [48]. There are several clinical trials that aim to deplete Tregs by targeting surface markers such as CD25, GITR, OX40, and CCR4 [115]. However, many do not show effective anti‐tumor responses. A possible explanation might be that some of these targeted surface markers are also important for immune effector functions. Thus, a better understanding of Treg biology and how to manipulate Treg metabolism and function with minimal effect on effector function within the TME has become a major focus of researchers’ intent on devising novel anticancer therapies.

### Treg metabolism in the TME

As described above, even in normal tissues Tregs and Teffs display metabolic differences that determine their fates. Inevitably, all types of T cells entering the TME have to undergo metabolic reprogramming to adapt to this harsh environment such as imbalanced metabolic nutrients, increased ROS and low oxygen level (hypoxia) [111, 116]. For example, in murine MC38 colorectal carcinomas model, intra‐tumoral Tregs invoke supplemental energy production routes involving increases in glycolysis and lipid metabolism [117]. This enhanced glucose and lipid uptake may fuel oxidative metabolism in a manner that confers a metabolic benefit and relative advantage on Tregs in the TME. This situation stands in stark contrast to the case in non‐cancerous tissues, where an intrinsic increase in glycolysis is linked to a reduction in FoxP3 and decreased Treg suppressive capacity [22, 61]. Once in the TME, Tregs are able to oxidize lactate into pyruvate when metabolic conditions are normal, or if a low glucose/high lactate environment prevails. In contrast, high lactate environment profoundly suppress effector T cell functions [48]. This adaptability of Tregs is due to FoxP3, which suppresses Myc and glycolysis and reprograms the metabolism of Tregs such that they can thrive in a low glucose/high lactate environment [57]. Moreover, Tregs are less susceptible to lactate overload than Teffs due to their decreased dependence on glycolysis and their generation of high levels of NAD during OXPHOS. These metabolic adaptations allow Tregs to carry out their task of promoting peripheral tolerance in the TME, disabling antitumor Teffs and thus perhaps partially explaining how cancer cells evade immune responses [57, 118].

Increased ROS level in the TME present an additional challenge for effective antitumor immunity. Several studies have shown the antagonistic effect of ROS on dampening Teff activity that results in deleterious effect on antitumor immunity [93, 111, 119]. Nevertheless, the effect of ROS on Treg biology within the TME is less clear. It has been shown that Treg contain higher thiol as well as antioxidant glutathione, which may provide Tregs the advantage against the oxidative microenvironment due to the accumulation ROS in the TME [61, 120]. Indeed, our group has recently shown that modulating ROS level in murine Treg result in an improved antitumor immunity [61]. Upon the ablation of glutathione in Tregs, these mice exhibited impaired Treg function and stronger anti‐tumor response upon the inoculation with B16F10 melanoma [61].

### Inhibition of Treg glycolysis or migration

Pretreatment of human Tregs *in vitro* with TLR8 agonist or 2‐DG to inhibit glycolysis impairs the Tregs’ ability to induce the senescence of CD8^+^ Teffs [121]. Interestingly, Kishore et al. showed that deletion of the glycolytic enzyme glucokinase in murine Tregs does not affect their suppressive function *in vitro*. However, glucokinase is critical for Treg trafficking and that glucokinase‐deficient Tregs do suffer from impairments to cytoskeletal rearrangement and actin remodeling that render them unable to migrate to the periphery [47]. Such a treatment in the cancer setting might provide the benefit of sustaining systemic immune tolerance, while at the same time blocking Treg migration to a tumor site and thereby preventing Tregs from dampening antitumor Teff responses. Evidence supporting this notion comes from a study of ovarian cancer patients in which both tumor cells and tumor‐associated macrophages were found to secrete the chemokine CCL2. CCL2 induces Tregs to preferentially migrate into the TME where they promote immune tolerance [122]. Thus, blocking metabolites that support Treg trafficking may help to preserve the functionality of anti‐tumor Teffs, determining the outcome of tumor growth in various cancer settings.

### Blocking HIF1α

Under hypoxic conditions (which are common in solid tumors), HIF1α is activated and orchestrates signaling cascades promoting cellular metabolism and survival. HIF1α is associated with control of the Th17/Treg balance, although its effect on Treg function is controversial [56, 123]. Several studies have reported that HIF1α activation positively drives the differentiation of FoxP3^+^ T cells both *in vitro* and *in vivo* [123, 124]. On the other hand, Dang et al. showed that HIF1α had an inhibitory effect that attenuated Treg differentiation while promoting Th17 induction *in vivo* [56]. Hsiao et al. have further shown that inhibition of HIF1α preserves Treg stability *in vitro*, although the suppressive ability is greatly impaired *in vivo* [125]. To clarify these discrepancies, Miska et al. deleted HIF1α specifically in murine Tregs and found that their suppressive capacity was enhanced *in vitro* [126]. They further showed that conditional deletion of HIF1α in Tregs restricts their glucose uptake, leaving them to use FA for the mitochondrial metabolism needed to support Treg function. On the other hand, in a mouse model of glioblastoma, mutant mice bearing HIF1α‐deficient Tregs showed better survival than controls due to impaired Treg migration to tumor sites [126]. Thus, HIF1α seems to play an important role in Tregs as a metabolic switch between glycolysis‐driven migration and FA‐dependent OXPHOS supporting Treg suppressive function.

### Manipulating FA

During tumorigenesis, free FA are released from more complex lipids, altering the FA composition of the TME [127]. Tregs in this setting express high levels of the FA transporters CD36 and SLC27A1, which allow these cells access to additional energetic sources to fuel FA metabolism [10, 128]. This exploitation of FA by Tregs in the TME provides the Tregs with the nutrients they need to survive and be fully functional, resulting in the barring of Teffs from attacking cancer cells. In addition, FA synthesis is enhanced in Tregs located in the TME, but not in those resident in the secondary lymphoid organs [117].

A recent study has shown that tumor‐infiltrating Tregs in mice exhibit higher glucose uptake. Higher glucose uptake has been shown to increase FA synthesis [117]. The increase in glycolysis and glycolytic‐derived FA pool lead to increased oxidative metabolism and support Treg expansion and proliferation without compromising Treg function, which dampen anti‐tumor immunity. *De novo* synthesized FA may be utilized not only to support cellular metabolism, but also to support histone acetylation and epigenetic reprogramming, an important factor for FoxP3 stability [129]. Likewise, Wang et al. showed that dampening FA metabolism via genetic deletion of CD36 in murine Tregs results specifically in lower numbers of tumor‐infiltrating Treg and decelerated tumor growth. Interestingly, the ablation of CD36 did not alter the homeostasis and functionality of splenic Treg cells [128]. This shows that FA metabolism via CD36 uniquely shape metabolic adaptation which supports the functionality of tumor‐infiltrating Treg cells.

In another study, inhibition of lipid uptake with sulfo‐*N*‐succinimidyl oleate (SSO) (Table 1) has been shown to significantly reduce the immunosuppressive capacity of Tregs *in vitro* [126]. Similarly, 5‐(tetradexyloxy)‐2‐furoic acid (TOFA), an inhibitor of ACC and thus FA synthesis (Table 1), significantly suppressed tumor development in mice [117]. TOFA's antitumor activity may be accounted for by its direct toxic effects on tumor cells (as evidenced by their reduced viability). However, *in vitro*, TOFA‐treated Tregs show a significantly decreased lipid pool and impaired proliferation, indicating that FA synthesis may play a vital role in Treg biology in this context [117]. Further studies are needed to elaborate whether the connections between FA metabolism in the TME and Tregs are in fact promoting tumor cell development, and whether this scenario could point to new strategies for cancer therapy.

### AA modulation

Manipulation of AA metabolism may serve as another mode of cancer immunotherapy, although most AAs are important for both tumor cells and adaptive immune cells [118]. As noted above, IDO metabolizes tryptophan to kynurenine, a metabolite important for Treg induction [88, 130]. Many types of cancer cells overexpress IDO, which might then drive Treg activity and so dampen anti‐tumor Teffs [18]. Cyclooxygenase2 (COX2) is involved in the upregulation of IDO expression in myeloid cells [131]. Accordingly, pharmacological inhibition of COX2 is under testing in various cancer models. For example, in a murine bladder tumor model, IDO inhibition reduced numbers of circulating Tregs and enhanced antitumor responses [132]. IDO is also implicated in Treg regulation because it stabilizes PTEN, which Tregs require to retain their functionality. PTEN dysregulation leads to aberrant mTOR activity, loss of Treg function, and spontaneous development of autoimmunity in mice [54]. In a mouse model of Lewis lung carcinoma, PTEN inhibition in Tregs plus IDO neutralization produced synergistic effects that greatly delayed disease relapse [133].

Lastly, we have linked the serine metabolism in Tregs to anti‐tumor immunity. The absence of GSH in Tregs and increased ROS levels, leads to enhanced serine metabolism and higher mTOR signaling, which in turn impairs tTreg and pTreg suppressive activity [61]. This defect in Tregs consequently increases anti‐tumor immunity and significantly reduces cancer cell growth *in vivo*.

Taken together, the various studies described above point to the possibility of altering key aspects of AA metabolism in Tregs to improve cancer immunotherapy.

## Concluding remarks

Our current understanding of the role of metabolism in immune regulation in general and in Tregs in particular is rapidly expanding, but many aspects remain to be addressed. We do know that Tregs are indispensable regulators of immune homeostasis. Accordingly, their dysregulation is implicated in numerous pathologies. The studies we have reviewed highlight the multifaceted relationships between the intrinsic and extrinsic metabolic pathways modulating Treg function, and their significant implications for the treatment of immune‐related diseases.

Tregs have a metabolic profile that is distinct from that of Teffs. Specific nutrients and metabolites obtained from the diet and/or extracellular milieu can influence Treg metabolism and affect their function. These factors also play important roles in Treg‐intrinsic metabolic regulation. Treg metabolism is highly plastic and adapts to the environmental context. Although their chief preference is FA utilization, Tregs are able to use various substrates to support their metabolism. Metabolic regulators such as mTOR and AMPK are indispensable for orchestrating Treg metabolism in several different ways, such that their dysregulation has differential effects on Treg functionality. For example, increased glycolysis leads to upregulation of mTOR and impaired Treg suppressive capacity, but also supports Treg migration.

Furthermore, it is also worth noting that the effects of pharmacological treatment *in vivo* may have dual action on both Teff and Treg simultaneously, in which the significance on one or the other cells still needs to be further evaluated.

Overall, although extensive studies on murine Treg have been conducted, significant challenges are still unsolved. There is Treg heterogeneity between subpopulations and their specific metabolic regulations remain to be explored. It is also important to acknowledge that that human and murine Tregs have different Treg subtypes such as the human type 1 regulatory T cells (Tr1), which only rely on glycolysis instead of fatty acid oxidation. Tr1 cells are also an important source of IL‐10 and TGF‐β [134]. Additionally, more work remains to be invested to evaluate the differences in metabolic requirement between similar subtypes of human and murine Treg; *in vitro* murine tTreg cells display low glycolysis and high FAO whereas human tTreg cells rely on high glycolysis and FAO [95].

In conclusion, modulating Treg metabolism may provide new tools that can be exploited in diverse disease settings. Inhibition of various metabolic enzymes has been suggested or is currently being studied in order to influence Treg function in AI diseases and cancer. Thus, novel Treg cell‐based immune metabolic interventions for the treatment of these disorders may prove to be beneficial in the near future.

## Conflict of interest

The authors declare no commercial or financial conflict of interest

**Abbreviations**
2‐DG: 2‐deoxy‐d‐glucose
AA: amino acid
ACC: acetyl‐CoA carboxylase
ACLY: ATP‐citrate lyase
AI: autoimmune
ATRA: all‐*trans* retinoic acid
BCAA: branched‐chain AA
CNS: conserved non‐coding DNA sequence
CPT‐1: carnitine palmitoyl transferase‐1
DCA: dichloroacetate
ETC: electron transport chain
FA: fatty acid
FAO: fatty acid oxidation
GGPP: geranylgeranylpyrophosphate
GSH: glutathione
GVHD: graft‐versus‐host disease
HMGCR: HMG‐CoA reductase
IBD: inflammatory bowel disease
LC‐FA: long‐chain fatty acid
LDH: lactate dehydrogenase
LKB1: liver kinase B1
OAA: oxaloacetate
OXPHOS: oxidative phosphorylation
PDH: pyruvate dehydrogenase
PP2A: protein phosphatase 2A
PPAR‐γ: Peroxisome proliferator‐activated receptor‐γ
PPP: pentose phosphate pathway
pTreg: peripherally derived Treg
RA: rheumatoid arthritis
SCFA: short‐chain fatty acid
SLE: systemic lupus erythematosus
TCR: T cell receptor
Th cell: T helper cell
TME: tumor microenvironment
TOFA: 5‐(tetradexyloxy)‐2‐furoic acid
Treg: regulatory T cell
TSC1: tuberous sclerosis 1
TSDR: Treg‐specific demethylation region
tTreg: thymus‐derived Treg
